# Prenatal exposure to extremely low frequency magnetic field and its impact on fetal growth

**DOI:** 10.1186/s12940-019-0447-9

**Published:** 2019-01-11

**Authors:** Yanfeng Ren, Jianping Chen, Maohua Miao, De-Kun Li, Hong Liang, Ziliang Wang, Fen Yang, Xiaowei Sun, Wei Yuan

**Affiliations:** 1 Department of Health Statistics, School of Public Health and Management, Weifang Medical University, Shandong, China; 2 Key Laboratory of Reproduction Regulation of NPFPC, SIPPR, IRD, Fudan University, 779 Old Hu Min Road, Shanghai, 200237 China; 3 Department of Fetal Medicine and Prenatal Diagnosis Center, Shanghai First Maternity and Infant Hospital, Tongji University School of Medicine, Shanghai, 200040 China; 4 Division of Research, Kaiser Foundation Research Institute, Kaiser Permanente, Oakland, California USA

**Keywords:** Magnetic field, Fetal growth, Cohort study, Pregnancy

## Abstract

**Objective:**

Studies on the effect of prenatal exposure to magnetic field (MF) on fetal growth is inconclusive and subject to some methodological limitations, particularly in measurement of MF exposure. The present study aimed to examine the association between maternal extremely low frequency MF (ELF-MF) exposure during pregnancy and fetal growth in offspring.

**Methods:**

A total of 128 pregnant women were recruited at their 3rd trimester and asked to wear an EMDEX Lite meter for 24 h to capture daily ELF-MF exposure. Time-weighted average (TWA), P50, and P75 of personal 24-h measurements were used to evaluate prenatal ELF-MF exposure. The medians of these measurements were used as cut-off points of high and low prenatal ELF-MF exposure. Fetal growth was measured by infant’s birth weight, skinfold thickness of triceps, abdomen, and back, and circumference of head, upper arm, and abdomen. These measures were conducted within 24-h after birth. Generalized Linear Model was used to examine the association between maternal ELF-MF level and fetal growth indices after potential confounders were adjusted for.

**Results:**

Compared with girls with lower prenatal ELF-MF exposure, girls with higher exposure had a lower birth weight, thinner skinfold of triceps, abdomen and back, and smaller circumference of head, upper arm and abdomen in all three ELF-MF matrices. The differences were statistically significant for birth weight and most other growth measurements (*P* < 0.05). These measures had no significant difference between higher and lower prenatal ELF-MF exposure in boys except back skinfold thickness.

**Conclusion:**

Prenatal exposure to higher ELF-MF levels was associated with decreased fetal growth in girls, but not in boys.

**Electronic supplementary material:**

The online version of this article (10.1186/s12940-019-0447-9) contains supplementary material, which is available to authorized users.

## Introduction

Magnetic field (MF) is a physical field produced by electrically charged objects and extends indefinitely throughout space by the electromagnetic interaction [1]. The introductions of electronic devices in daily life, such as communications and data transmission, household appliances, personal wireless devices, industry applications, and medical purposes, have led to ubiquitous exposure to MF for human being. Environmental MF exposure consists of two types of MFs: (a) power frequency or extremely low frequency (ELF-MF); and (b) radiofrequency/microwave radiation emissions (from wireless devices such as cordless and cell phones, cellular antennas, and broadcast transmission towers). There have been growing concerns regarding the potential health impacts of MF, ever since the first report on the increased risk of childhood leukemia for children living near power transmission lines in 1979 [2]. From then on, higher MF exposure level has been associated with childhood cancer, impairments in cognitive function, diabetes, poor sperm quality, reduced fertility, and other health outcomes [3–6]. As developing embryo and fetus are much more susceptible to environmental toxins including MF, many studies have focused on the impact of prenatal MF exposure on fetal growth as well as other health outcomes of offspring, such as miscarriage, obesity, asthma, etc. [3, 4, 7, 8].

Birth weight is a strong predictor of infants’ mortality/morbidity and has been regarded as a convenient surrogate for fetal growth [9]. Several studies have examined the associations between prenatal ELF-MF exposure and low birth weight (LBW), small for gestational age (SGA), or intrauterine growth retardation (IUGR) in offspring [10–12], with inconsistent results. One study investigated the relationships between ELF-MF exposure and other fetal growth indices including fetal length and head circumference, but no association was found [13] .

One of the main challenges in evaluating potential health impacts of MF exposure is to accurately measure personal MF exposure level. Most previous studies used wire code classification of residence, residential proximity to MF sources, or residential spot measurement by surrogate [10–12], which could lead to severe misclassification of personal MF measurement and bias the results towards null in most cases. One study added a 7-day personal MF measurement; however, the device used in the study was single-axis [11], which might not be able to reflect the ‘actual’ exposure level of an individual since the MF comes from multiple directions [14].

In the present study, we examined the association between maternal 24-h MF exposure at individual level, measured by a triple-axis device during the 3rd trimester, and fetal growth in offspring in a prospective cohort study.

## Methods

### Study population

This study was based on data from the Shanghai-Minhang Birth Cohort (S-MBC). The cohort was established to determine the environmental exposures among pregnant women and to explore the impacts of those exposures on pregnant women and their offspring. A total of 1292 pregnant women at gestational age of 12th–16th weeks in Maternal and Child Health Hospital of Minhang District, Shanghai were recruited into the cohort study between April and December 2012. Women were eligible for recruitment if they: 1) were residents of Shanghai; 2) did not have serious chronic diseases; 3) carried singleton pregnancy; 4) planned to carry their pregnancies to term; and 5) were willing to complete the questionnaires. We invited 250 pregnant women from the cohort to participate in the present study at gestational age of 28th–36th weeks, due to the limited MF measuring instruments. Among them 140 women participated and provided their written informed consent. During the study period, 12 of them didn’t complete the 24-h measurement as required, and the remained 128 live birth babies were included in the present analyses.

### Measurement of personal magnetic field exposure

EMDEX Lite meter (Enertech Consultants, Campbell, CA, USA) was used to measure personal MF exposure level. The device collects MF measurements from triple-axis in milligauss (1mG = 0.1 μt) in the extremely low frequency band ranging from 40 to 1000 Hz, which covers the dominant MF of 50 Hz power-line frequency in China. The meter has been widely used in epidemiologic studies [5, 8, 15].

MF level of each participating woman was monitored consecutively for 24 h in a typical day of the recent 3 months. Participants were asked to wear the meter at the waist level in daytime and to place it next to beds while sleeping. They were also asked to keep a diary record for all her activities in the 24 h of the measurement period. The meter was programmed to measure and store the MF every 4 s, and MF levels were not displayed so that the women would not change their regular activities accordingly. Time-weighted average (TWA), median (P50) and 75th percentile (P75) of the 24-h MF measures of each woman were calculated. Time-weighted average (TWA) was defined as the time-weighted exposure intensity. P50 was defined as the MF level above which 50% of all 24-h measures had a higher value, which corresponded to 12 h of higher exposure during the measurement period. P75 was defined as the MF level above which 25% of all 24-h measurements had a higher value, which corresponded to 6 h of higher exposure during measurement period.

### Outcome and potential confounders

The indices of fetal growth used in the present study included birth weight, circumference of head, upper arm, and abdomen, and skinfold thickness of back, triceps, and abdomen. Birth length was not included in our study since it is not a practically good measure of fetal growth because of the difficulty and inaccuracy in measuring and the small variation across newborns. Information on birth weight was obtained from the medical records immediately after birth. Head, upper arm and abdominal circumferences were measured in centimeters using a flexible tape. Head circumference was measured on a line passing over the glabella and the posterior occipital protrusion. Abdominal circumference was accurately measured along a line at the level of the umbilicus. Upper arm circumference was measured at the midway between the olecranon and acromial process on the upper right arm. Skinfold thickness was measured by a caliper (Holtain, Crymych, UK), with a sensitivity of 0.1 mm. Triceps skinfold thickness is measured in the posterior midline of the right arm, over the triceps midway between the acromion process and the olecranon process. Back skinfold thickness is measured immediately below the tip of the inferior angle of the right scapula at an angle of 45-degree with vertical axis. Abdominal skinfold thickness is measured at the site of 1 cm from umbilicus to the right. Two trained physicians conducted all measurements. A child was measured twice by the two physicians and the averages of their readings were used for analysis.

Demographic characteristics of pregnant women and their spouses, including pre-pregnant weight and height, socio-economic status, and passive smoking during pregnancy were collected face to face using a structured questionnaire by trained interviewers at recruitment. Body mass index (BMI) was calculated as body weight in kilograms divided by squared body height in meters. After delivery, information on sex and parity of the child was obtained from the medical records.

### Statistical analysis

MF exposure by characteristics of the pregnant women was presented as medians and interquartile ranges (IQR) of the TWA, P50 and P75 of the MF measures. The medians were also used as cutoff points of higher and lower MF exposure groups. Birth weight, skinfold thickness of back, triceps and abdomen as well as circumference of head, upper arm, and abdomen were compared across MF exposure groups using generalised linear model (GLM). Demographic factors, including gestational age, parity, maternal BMI before pregnancy, and maternal passive smoking during pregnancy, maternal and paternal ages at childbirth, paternal BMI, and family income were adjusted for as potential confounders. All the adjusted variables were included in the model.

Since previous study indicated that MF might have gender-specific effects [12], stratified analyses by infant’s sex were performed in addition to examining the overall associations. To test the robustness of the results, we repeated the analyses among termed birth babies and babies whose mothers didn’t experience gestational diabetes. As the level of MF exposure was significantly different among maternal age in our study, we further stratified analysis to test the consistence of the association between maternal MF exposure and fetal growth by maternal age. All analyses were conducted in SAS software, version 9.1 (SAS Institute).

## Results

### Exposure level by characteristics of the study patients

Among 128 newborns, girls and boys accounted for 50.0%, respectively. The mean birth weight was 3480 g for boys and 3440 g for girls. Only two children were born with LBW(< 2500 g), two children were born preterm, and five children had mothers who suffered from diabetes mellitus, including two gestational diabetes mellitus.

Table 1 shows the median and interquartile ranges of TWA, P50 and P75 according to characteristics of pregnant women. Compared with the group of “women’s age <25 years”, TWA and P75 were significantly higher in the group of “women’s age ≥30 years”. Women with BMI of “≥24 kg/m^2^” or unexposed to passive smoking during pregnancy had higher TWA, P50 and P75. Pregnant women with average family income level of “< 2000 RMB” were more likely to be exposed to higher TWA, P50 and P75 than those with higher family income levels. Pregnant women who engaged in mental labor were more likely to be exposed to lower TWA, P50 and P75 than those who engaged in manual labor. Compared with women with female infant, TWA, P50 and P75 were higher among those with male infant.

**Table 1:** Characteristics of the women in relation to ELF-MF parameters

Characteristics	N	Median (Q1, Q3)(mG)		
		TWA	P50	P75
Total	128	0.63 (0.38,1.09)	0.38 (0.23,0.68)	0.63 (0.38,1.17)
Maternal age at child birth (years)				
<25	14	0.45 (0.28,0.63)^*^	0.36 (0.18,0.48)	0.50 (0.33,0.68)^*^
25–30	79	0.64 (0.38,1.04)	0.38 (0.23,0.63)	0.63 (0.38,1.17)
> 30	35	0.71 (0.45,1.49)	0.48 (0.23,0.82)	0.82 (0.38,1.52)
Maternal BMI before pregnancy (kg/m^2^)				
<18.5	28	0.64 (0.41,1.11)	0.41 (0.28,0.70)	0.66 (0.41,1.15)
18.5–24	93	0.58 (0.37,1.04)	0.38 (0.18,0.63)	0.57 (0.33,1.17)
> 24	7	1.22 (0.39,1.53)	0.82 (0.23,0.97)	1.13 (0.48,1.52)
Maternal education level				
Middle school and below	7	0.66 (0.58,0.90)	0.38 (0.33,0.48)	0.57 (0.57,0.77)
High school	15	0.75 (0.35,1.56)	0.43 (0.18,0.77)	0.68 (0.33,1.73)
College and undergraduate	96	0.64 (0.39,1.10)	0.43 (0.23,0.72)	0.66 (0.38,1.17)
Postgraduate and above	10	0.33 (0.23,0.80)	0.21 (0.13,0.52)	0.33 (0.23,0.88)
Maternal employment				
Manual labor	84	0.64 (0.38,1.06)	0.43 (0.23,0.70)	0.66 (0.38,1.17)
Mental labor	30	0.49 (0.37,0.90)	0.36 (0.23,0.52)	0.55 (0.43,0.88)
Others	14	0.79 (0.57,1.36)	0.56 (0.33,1.17)	0.88 (0.57,1.52)
Average family income / month^*^person (RMB)				
< 4000	28	0.70 (0.44,1.40)	0.48 (0.28,0.80)	0.70 (0.43,1.68)
4000~10,000	75	0.58 (0.38,1.07)	0.38 (0.23,0.63)	0.63 (0.38,1.13)
> 10,000~	24	0.64 (0.32,1.05)	0.31 (0.18,0.62)	0.67 (0.38,1.33)
Maternal passive smoking				
Yes	56	0.53 (0.37,0.88)	0.36 (0.21,0.68)	0.52 (0.36,1.03)
No	69	0.67 (0.40,1.10)	0.43 (0.23,0.63)	0.77 (0.43,1.48)
Parity				
1	111	0.63 (0.38,1.10)	0.43 (0.23,0.68)	0.63 (0.38,1.17)
≥ 2	17	0.69 (0.25,1.08)	0.33 (0.18,0.63)	0.57 (0.33,1.17)
Infant sex				
Male	64	0.65 (0.40,1.29)	0.40 (0.25,0.79)	0.72 (0.40,1.52)
Female	64	0.54 (0.37,0.89)	0.38 (0.20,0.65)	0.57 (0.38,0.93)

*Abbreviations*: *TWA* time-weighted average

^*^Kruskal-Wallis Test: *P* < 0.05

### Effects of MF exposure on birth weight

Newborns with higher prenatal MF exposure had significantly lower birth weight in all MF exposure measure matrices (TWA, P50 and P75) compared to those who exposed to lower levels. The association remained statistically significant after adjusting for potential confounders including gestational age, parity, maternal BMI before pregnancy, and maternal passive smoking during pregnancy, maternal and paternal ages at childbirth, paternal BMI, and family income. Further stratified analysis showed that the associations were only observed among girls, but not among boys (Table 2).

**Table 2:** Relationship between in-utero ELF-MF exposure and birth weight (grams) using GLM

MF measurements	All				Boys				Girls			
	N	$\overline{x}\pm \mathrm{s}$	P	P^#^	N	$\overline{x}\pm \mathrm{s}$	P	P^#^	N	$\overline{x}\pm \mathrm{s}$	P	P^#^
TWA												
Below median	63	3547.7 ± 440.5	0.02	0.02	29	3471.3 ± 427.7	0.89	0.75	34	3612.9 ± 447.0	< 0.01	< 0.01
Above median	65	3374.7 ± 393.0			35	3486.8 ± 425.7			30	3244.0 ± 309.2		
P50												
Below median	55	3548.0 ± 455.0	0.04	0.01	26	3456.1 ± 428.8	0.71	0.47	29	3630.3 ± 469.3	< 0.01	< 0.01
bove median	73	3393.5 ± 389.9			38	3496.0 ± 424.5			35	3282.2 ± 318.5		
P75												
Below median	62	3538.2 ± 446.8	0.04	0.02	26	3461.2 ± 451.5	0.77	0.48	36	3593.8 ± 441.3	< 0.01	< 0.01
Above median	66	3386.3 ± 391.4			38	3492.6 ± 408.6			28	3242.1 ± 320.4		

^#^Adjusting for family income, gestational age, parity, parental age at childbirth, parental BMI before pregnancy, and maternal passive smoking during pregnancy

### Effects of MF exposure on skinfold thickness

Newborns with higher prenatal exposure measurements of TWA, P50 and P75 had thinner abdominal and back skinfold compared with those with lower prenatal MF exposure levels, although the difference of abdominal skinfold thicknesses was not statistically significant between the groups below and above the median levels of exposure. Similarly, the associations were only observed for girls, but not for boys. Similar pattern was observed for triceps skinfold, but the difference was of borderline significance in the exposure measurement of P75 (*P* = 0.07) (Table 3).

**Table 3:** Relationship between in-utero ELF-MF exposure and skinfold thickness (cm) using GLM

MF measurements		All				Boys				Girls			
		N	$\overline{x}\pm \mathrm{s}$	P	P^#^	N	$\overline{x}\pm \mathrm{s}$	P	P^#^	N	$\overline{x}\pm \mathrm{s}$	P	P^#^
Abdominal skinfold thicknesses	TWA												
	Below median	63	2.8 ± 0.8	0.12	0.09	29	2.6 ± 0.7	0.84	0.86	34	2.9 ± 0.9	0.09	0.02
	Above median	65	2.5 ± 0.6			35	2.5 ± 0.7			30	2.5 ± 0.6		
	P50												
	Below median	55	2.8 ± 0.9	0.22	0.22	26	2.4 ± 0.7	0.25	0.17	29	3.0 ± 0.9	0.02	0.01
	Above median	73	2.6 ± 0.6			38	2.6 ± 0.6			35	2.5 ± 0.6		
	P75												
	Below median	62	2.7 ± 0.9	0.23	0.15	26	2.5 ± 0.8	0.46	0.28	36	2.9 ± 0.9	0.06	0.02
	Above median	66	2.6 ± 0.6			38	2.6 ± 0.6			28	2.5 ± 0.6		
Triceps skinfold thickness	TWA												
	Below median	63	4.4 ± 1.2	0.02	0.02	29	4.2 ± 1.1	0.15	0.09	34	4.5 ± 1.3	0.06	0.04
	Above median	65	3.8 ± 1.1			35	3.8 ± 1.1			30	3.8 ± 1.2		
	P50												
	Below median	55	4.4 ± 1.2	0.02	0.03	26	4.1 ± 1.1	0.76	0.44	29	4.7 ± 1.3	< 0.01	0.01
	Above median	73	3.9 ± 1.1			38	4.0 ± 1.1			35	3.7 ± 1.1		
	P75												
	Below median	62	4.3 ± 1.2	0.13	0.09	26	4.0 ± 1.2	0.96	0.69	36	4.5 ± 1.3	0.07	0.07
	Above median	66	3.9 ± 1.1			38	4.0 ± 1.0			28	3.8 ± 1.2		
Back skinfold thickness	TWA												
	Below median	63	4.4 ± 1.2	< 0.01	< 0.01	29	4.3 ± 1.1	0.02	0.03	34	4.5 ± 1.3	< 0.01	< 0.01
	Above median	65	3.6 ± 0.8			35	3.7 ± 0.9			30	3.6 ± 0.8		
	P50												
	Below median	55	4.4 ± 1.3	< 0.01	0.01	26	4.2 ± 1.3	0.14	0.21	29	4.5 ± 1.3	< 0.01	< 0.01
	Above median	73	3.8 ± 0.8			38	3.8 ± 0.8			35	3.7 ± 0.8		
	P75												
	Below median	62	4.3 ± 1.2	< 0.01	0.02	26	4.1 ± 1.3	0.34	0.54	36	4.5 ± 1.2	< 0.01	< 0.01
	Above median	66	3.7 ± 0.8			38	3.9 ± 0.8			28	3.5 ± 0.7		

^#^Adjusting for family income, gestational age, parity, parental age at childbirth, parental BMI before pregnancy, and maternal passive smoking during pregnancy

### Effects of MF exposure on circumference of head, up-arm, and abdomen

Similar patterns were also observed for head, upper arm, and abdominal circumference, with statistically or marginally significant differences of the above circumferences between girls with higher and lower prenatal MF exposure in all MF exposure measure matrices (TWA, P50 and P75) (Table 4).

**Table 4:** Relationship between in-utero ELF-MF exposure and circumference of head, up arm and abdomen (cm) using GLM

MF measurements		All				Boys				Girls			
		N	$\overline{x}\pm \mathrm{s}$	P	P^#^	N	$\overline{x}\pm \mathrm{s}$	P	P^#^	N	$\overline{x}\pm \mathrm{s}$	P	P^#^
Head circumference	TWA												
	Below median	63	35.3 ± 1.2	0.13	0.16	29	35.5 ± 1.2	0.44	0.42	34	35.1 ± 1.1	0.07	0.08
	Above median	65	34.9 ± 1.3			35	35.2 ± 1.5			30	34.6 ± 0.9		
	P50												
	Below median	55	35.3 ± 1.2	0.12	0.06	26	35.5 ± 1.3	0.65	0.18	29	35.2 ± 1.1	0.02	0.02
	Above median	73	34.9 ± 1.3			38	35.3 ± 1.5			35	34.5 ± 0.9		
	P75												
	Below median	62	35.3 ± 1.2	0.18	0.08	26	35.5 ± 1.3	0.53	0.16	36	35.1 ± 1.1	0.04	0.06
	Above median	66	34.9 ± 1.3			38	35.3 ± 1.4			28	34.5 ± 0.9		
Upper arm circumference	TWA												
	Below median	63	11.3 ± 1.2	0.01	0.03	29	11.1 ± 1.1	0.26	0.17	34	11.5 ± 1.4	0.0262	0.05
	Above median	65	10.8 ± 0.9			35	10.8 ± 1.1			30	10.8 ± 0.7		
	P50												
	Below median	55	11.3 ± 1.3	0.03	0.03	26	11.1 ± 1.1	0.54	0.30	29	11.6 ± 1.5	0.0146	0.02
	Above median	73	10.9 ± 0.9			38	10.9 ± 1.1			35	10.8 ± 0.6		
	P75												
	Below median	62	11.3 ± 1.3	0.03	0.02	26	11.1 ± 1.1	0.57	0.26	36	11.5 ± 1.3	0.0200	< 0.05
	Above median	66	10.8 ± 0.9			38	10.9 ± 1.1			28	10.8 ± 0.7		
Abdominal circumference	TWA												
	Below median	63	33.9 ± 2.0	0.10	0.03	29	33.6 ± 2.2	0.87	0.54	34	34.3 ± 1.9	< 0.01	0.03
	Above median	65	33.3 ± 1.8			35	33.7 ± 1.9			30	32.9 ± 1.6		
	P50												
	Below median	55	33.9 ± 2.1	0.24	0.11	26	33.5 ± 2.2	0.72	0.55	29	34.2 ± 2.1	0.03	0.04
	Above median	73	33.4 ± 1.8			38	33.7 ± 2.0			35	33.1 ± 1.5		
	P75												
	Below median	62	33.9 ± 2.0	0.19	< 0.05	26	33.6 ± 2.2	0.86	0.34	36	34.1 ± 1.9	0.03	0.06
	Above median	66	33.4 ± 1.8			38	33.7 ± 2.0			28	33.0 ± 1.6		

^#^Adjusting for family income, gestational age, parity, parental age at childbirth, parental BMI before pregnancy, and maternal passive smoking during pregnancy

### Sensitivity analysis of effects of MF exposure on fetal growth

We repeated the analysis among the babies who were carried to term birth or whose mothers didn’t experience gestational diabetes, but the results didn’t change substantially (Data no shown).

In addition, stratified analysis by maternal age showed generally similar pattern, although the sample size for those with maternal age < 25 is too small to produce reliable results (Additional file 1: Table S1, Table S2 and Table S3).

## Discussion

In the present prospective cohort study, we found that inverse associations between prenatal ELF-MF exposure and fetal growth were only in girls. Compared with girls whose prenatal MF level was below median, girls with prenatal MF level above medians of TWA, P50 and P75 had a lower birth weight, thinner skinfolds of back, triceps, and abdomen, and smaller circumferences of head, up-arm, and abdomen. The results indicated a sex specific effect of prenatal ELF-MF on fetal growth.

Our study was supported by several previous findings [12, 16], but not all. Vocht F et al. found a decreased birth weight for living close (≤50 m) to ELF-MF sources compared with those living > 200 m during pregnancy, and the decrease was larger for female than male babies(− 251 g vs -137 g) [12] . Another study found a 5% increased risk of SGA for living < 50 m from transmission lines, compared to those living ≥400 m [10]. A cross-sectional study on 780 children aged between 0 and 12 years found that exposure to ELF-MF from high voltage electric power lines was associated with suboptimal growth profile of children, including lower circumferences of the head, chest and height at all studied ages [16]. However, one previous study showed no difference on the average birth weight following exposure to electric heated beds during pregnancy [11]. Another study did not find significant association between exposure to high voltage electricity towers and cables and neonatal head circumference [13]. The difference among these studies might lie in the ability to accurately measure actual ELF-MF exposure during pregnancy. Most previous studies used indirect prenatal MF measurements [10–12] and the only study which measured a 7-day MF exposure used a single-axis device rather than a triple-axis device [11], all of which might compromise the accuracy of MF measurements and therefore led to misclassification of prenatal MF exposure. Such misclassification generally led to attenuation of an underlying MF effect because of the more likely nature of non-differential. Moreover, gender differences were not well addressed in some studies and the confounding could not be appropriately considered.

Although the biological mechanisms of the effect of prenatal ELF-MF exposure on fetal growth are currently unclear, ELF-MF has been reported to be able to influence the oxidated state and intracellular Ca^2+^ signaling patterns at a cellular level [17, 18], which could in turn result in placental vascular function changes and subsequently suboptimal fetal growth [19]. Another study showed that MF exposure could impact cellular glucose activity level [20], which provided another possible mechanism of our observed associations. In addition, we had previously reported associations between maternal ELF-MF exposure and increased risk of shorter embryonic bud length, miscarriage, and obesity. Therefore, it is biologically plausible that prenatal ELF-MF exposure have an adverse effect on fetal growth.

The study had several strengths. The most important strength was the prospective nature of the study design. Information on prenatal ELF-MF exposure was collected before child birth, which reduced information bias compared to measurements done retrospectively. Second, we measured actual personal ELF-MF exposure with measuring instrument quantitatively, which provided a more accurate ELF-MF measurement and thus a better opportunity to detect the association between prenatal ELF-MF exposure and fetal growth, if existed. Third, the present study was able to control for a number of potential confounders such as parental BMI before pregnancy [21] and maternal passive smoking during pregnancy [22], which were reported to play an important role in birth weight.

Several potential limitations should be kept in mind when the results of this study were interpreted. First, the single 24-h measurement might limit the capability to acquire the complete profile of ELF-MF exposure during the time period of interest, resulting in misclassification of the MF exposure level. However, the design that woman wore meter in a typical day and was monitored consecutively for 24 h, increased measurement validity of subjects’ exposure during the 3rd trimester. In addition, the misclassification would most likely be non-differential in prospective study, and would bias the results towards null. Moreover, the measurement of MF in a typical day might reduce the potential misclassification to a large extent [23]. Second, only MF exposure from extremely low frequency was collected by EMDEX Lite meter; Thus, our finding may or may not apply to MF in other frequencies. Third, the participation rate was somewhat low in the study (about 56%). It was a prospective cohort study and MF exposure level was largely unknown to the general public, so that the low participation rate was unlikely to be associated with MF exposure. Fourth, a chance finding could exist since the sample size in boys or girls was relatively small. However, the consistent associations observed across various MF matrices (TWA, P50, and P75) and fetal growth indices might alleviate the concern. Fifth, the information of maternal smoking during pregnancy was not collected. However, only about 2.4% of women smoke in China [24]. The percentage of women who smoked during pregnancy was expected to be lower than 2.4% in this cohort of pregnant women and was not expected to bias our estimates largely. Alternatively, passive smoking might play a more important role in confounding our results and had already been adjusted for throughout the analyses. Finally, the small sample size did not allow evaluating the dose-responsive effect with finer categories of exposure, and restricted the ability to verify the associations by high exposure. Further prospective cohort studies with larger sample size will be more promising to verify the potential adverse effect of prenatal exposure to MF.

In conclusion, this study indicated that prenatal exposure to higher ELF-MF could result in reduction of birth weight, circumference of head, upper arm, and abdomen and skinfold thickness of back, triceps and abdomen in girls, but not in boys. Future studies with larger sample sizes are warranted to confirm our findings.

## Additional file

Additional file 1: **Table S1.** Relationship between in-utero ELF-MF exposure and birth weight (grams) using GLM. **Table S2.** Relationship between in-utero ELF-MF exposure and skinfold thickness (cm) using GLM. **Table S3.** Relationship between in-utero ELF-MF exposure and circumference of head, upper arm and abdomen (cm) using GLM. (DOCX 45 kb)
