# Supplementary material for: Prenatal exposure to extremely low frequency magnetic field and its impact on fetal growth
Source: Environ Health. 2019 Jan 11;18:6. doi: 10.1186/s12940-019-0447-9 (PMC6329146; doi:10.1186/s12940-019-0447-9)
Supplement: Supplementary file 1 — Table S1. Relationship between in-utero ELF-MF exposure and birth weight (grams) using GLM. Table S2. Relationship between in-utero ELF-MF exposure and skinfold thickness (cm) using GLM. Table S3. Relationship between in-utero ELF-MF exposure and circumference of head, upper arm and abdomen (cm) using GLM. (DOCX 45 kb) [file 12940_2019_447_MOESM1_ESM.docx]

Table S1 Relationship between in-utero ELF-MF exposure and birth weight (grams) using GLM

| MF measurements | Mother’ age<25 | | | | Mother’age 25-30 | | | | Mother’age>=30 | | | |
| --- | --- | --- | --- | --- | --- | --- | --- | --- | --- | --- | --- | --- |
|  | N | $\bar{x}\pm\text{s}$  | P | P^#^ | N | $\bar{x}\pm\text{s}$ | P | P^#^ | N | $\bar{x}\pm\text{s}$ | P | P^#^ |
| TWA |  |  |  |  |  |  |  |  |  |  |  |  |
| Below median | 10 | 3437.0±417.6 | 0.54 | 0.63 | 39 | 3559.2±446.8 | 0.06 | 0.04 | 14 | 3595.0±457.3 | 0.14 | 0.30 |
| Above median | 4 | 3285.0±388.2 |  |  | 40 | 3373.5±426.9 |  |  | 21 | 3394.3±338.3 |  |  |
| P50 |  |  |  |  |  |  |  |  |  |  |  |  |
| Below median | 7 | 3522.9±450.9 | 0.24 | 0.24 | 36 | 3556.9±451.3 | 0.09 | 003 | 12 | 3535.8±506.6 | 0.51 | 0.80 |
| Above median | 7 | 3264.3±325.1 |  |  | 43 | 3388.4±427.9 |  |  | 23 | 3442.6±334.0 |  |  |
| P75 |  |  |  |  |  |  |  |  |  |  |  |  |
| Below median | 10 | 3437.0±417.6 | 0.54 | 0.58 | 39 | 3549.5±449.8 | 0.09 | 0.01 | 13 | 3582.3±482.2 | 0.22 | 0.72 |
| Above median | 4 | 3285.0±388.2 |  |  | 40 | 3383.0±427.9 |  |  | 22 | 3410.9±332.0 |  |  |

^#^ Adjusting for family income, gestational age, parity, parental age at childbirth, parental BMI before pregnancy, and maternal passive smoking during pregnancy.

Table S2 Relationship between in-utero ELF-MF exposure and skinfold thickness (cm) using GLM.

| MF measurements | | Mother’ age<25 | | | | Mother’age 25-30 | | | | Mother’age>=30 | | | |
| --- | --- | --- | --- | --- | --- | --- | --- | --- | --- | --- | --- | --- | --- |
|  |  | N | $\bar{x}\pm\text{s}$  | P | P^#^ | N | $\bar{x}\pm\text{s}$ | P | P^#^ | N | $\bar{x}\pm\text{s}$ | P | P^#^ |
| **Abdominal skinfold thicknesses** | TWA |  |  |  |  |  |  |  |  |  |  |  |  |
|  | Below median | 10 | 2.7±0.8 | 0.83 | 0.52 | 39 | 2.7±0.8 | 0.14 | 0.09 | 14 | 2.9±0.8 | 0.39 | 0.26 |
|  | Above median | 4 | 2.8±0.3 |  |  | 40 | 2.5±0.7 |  |  | 21 | 2.6±0.6 |  |  |
|  | P50 |  |  |  |  |  |  |  |  |  |  |  |  |
|  | Below median | 7 | 2.9±0.8 | 0.36 | 0.11 | 36 | 2.7±0.9 | 0.56 | 0.49 | 12 | 2.0±0.9 | 0.31 | 0.66 |
|  | Above median | 7 | 2.6±0.6 |  |  | 43 | 2.6±0.7 |  |  | 23 | 2.6±0.6 |  |  |
|  | P75 |  |  |  |  |  |  |  |  |  |  |  |  |
|  | Below median | 10 | 2.7±0.8 | 0.83 | 0.52 | 39 | 2.7±0.9 | 0.33 | 0.32 | 13 | 2.9±0.9 | 0.31 | 0.42 |
|  | Above median | 4 | 2.8±0.3 |  |  | 40 | 2.5±0.6 |  |  | 22 | 2.6±0.6 |  |  |
| **Triceps skinfold thickness** | TWA |  |  |  |  |  |  |  |  |  |  |  |  |
|  | Below median | 10 | 3.9±1.0 | 0.60 | 0.70 | 39 | 4.5±1.3 | 0.03 | 0.05 | 14 | 4.4±1.1 | 0.09 | 0.13 |
|  | Above median | 4 | 4.2±0.3 |  |  | 40 | 3.9±1.3 |  |  | 21 | 3.7±0.9 |  |  |
|  | P50 |  |  |  |  |  |  |  |  |  |  |  |  |
|  | Below median | 7 | 4.1±1.1 | 0.53 | 0.27 | 36 | 4.4±1.3 | 0.14 | 0.13 | 12 | 4.5±1.2 | 0.05 | 0.36 |
|  | Above median | 7 | 3.8±0.7 |  |  | 43 | 4.0±1.3 |  |  | 23 | 3.7±0.8 |  |  |
|  | P75 |  |  |  |  |  |  |  |  |  |  |  |  |
|  | Below median | 10 | 3.9±1.0 | 0.60 | 0.70 | 39 | 4.4±1.3 | 0.26 | 0.17 | 13 | 4.4±1.2 | 0.12 | 0.39 |
|  | Above median | 4 | 4.2±0.3 |  |  | 40 | 4.0±1.3 |  |  | 22 | 3.8±0.9 |  |  |
| **Back skinfold thickness** | TWA |  |  |  |  |  |  |  |  |  |  |  |  |
|  | Below median | 10 | 3.9±0.9 | 0.29 | 0.38 | 39 | 4.6±1.3 | <0.01 | <0.01 | 14 | 4.3±1.0 | 0.04 | 0.27 |
|  | Above median | 4 | 4.5±0.5 |  |  | 40 | 3.6±0.9 |  |  | 21 | 3.6±0.6 |  |  |
|  | P50 |  |  |  |  |  |  |  |  |  |  |  |  |
|  | Below median | 7 | 4.1±0.9 | 0.83 | 0.98 | 36 | 4.5±1.4 | <0.01 | 0.02 | 12 | 4.2±1.1 | 0.13 | 0.65 |
|  | Above median | 7 | 4.0±0.7 |  |  | 43 | 3.8±0.9 |  |  | 23 | 3.7±0.6 |  |  |
|  | P75 |  |  |  |  |  |  |  |  |  |  |  |  |
|  | Below median | 10 | 3.9±0.9 | 0.29 | 0.38 | 39 | 4.4±1.4 | 0.01 | 0.03 | 13 | 4.3±1.1 | 0.05 | 0.34 |
|  | Above median | 4 | 4.5±0.5 |  |  | 40 | 3.7±0.9 |  |  | 22 | 3.6±0.7 |  |  |

^#^ Adjusting for family income, gestational age, parity, parental age at childbirth, parental BMI before pregnancy, and maternal passive smoking during pregnancy.

Table S3 Relationship between in-utero ELF-MF exposure and circumference of head, upper arm and abdomen (cm) using GLM

| MF measurements | | Mother’ age<25 | | | | Mother’age 25-30 | | | | Mother’age>=30 | | | |
| --- | --- | --- | --- | --- | --- | --- | --- | --- | --- | --- | --- | --- | --- |
|  |  | N | $\bar{x}\pm\text{s}$  | P | P^#^ | N | $\bar{x}\pm\text{s}$ | P | P^#^ | N | $\bar{x}\pm\text{s}$ | P | P^#^ |
| **Head circumference** | TWA |  |  |  |  |  |  |  |  |  |  |  |  |
|  | Below median | 10 | 34.6±0.9 | 0.71 | 0.57 | 39 | 35.3±1.1 | 0.39 | 0.62 | 14 | 35.7±1.5 | 0.07 | 0.15 |
|  | Above median | 4 | 34.4±1.2 |  |  | 40 | 35.1±1.4 |  |  | 21 | 34.8±1.2 |  |  |
|  | P50 |  |  |  |  |  |  |  |  |  |  |  |  |
|  | Below median | 7 | 34.9±0.7 | 0.13 | 0.29 | 36 | 35.4±1.1 | 0.29 | 0.30 | 12 | 35.4±1.7 | 0.42 | 0.77 |
|  | Above median | 7 | 34.2±1.1 |  |  | 43 | 35.1±1.3 |  |  | 23 | 35.0±1.2 |  |  |
|  | P75 |  |  |  |  |  |  |  |  |  |  |  |  |
|  | Below median | 10 | 34.6±0.9 | 0.71 | 0.57 | 39 | 35.4±1.1 | 0.27 | 0.20 | 13 | 35.6±1.6 | 0.21 | 0.44 |
|  | Above median | 4 | 34.4±1.2 |  |  | 40 | 35.0±1.4 |  |  | 22 | 34.9±1.2 |  |  |
| **Upper arm circumference** | TWA |  |  |  |  |  |  |  |  |  |  |  |  |
|  | Below median | 10 | 11.0±1.1 | 0.32 | 0.25 | 39 | 11.5±1.3 | 0.01 | 0.02 | 14 | 11.0±1.2 | 0.73 | 0.91 |
|  | Above median | 4 | 10.3±0.8 |  |  | 40 | 10.8±0.9 |  |  | 21 | 10.9±0.9 |  |  |
|  | P50 |  |  |  |  |  |  |  |  |  |  |  |  |
|  | Below median | 7 | 11.2±1.2 | 0.21 | 0.01 | 36 | 11.5±1.4 | 0.03 | 0.05 | 12 | 11.0±1.3 | 0.92 | 0.61 |
|  | Above median | 7 | 10.5±0.5 |  |  | 43 | 10.9±0.9 |  |  | 23 | 10.9±0.9 |  |  |
|  | P75 |  |  |  |  |  |  |  |  |  |  |  |  |
|  | Below median | 10 | 11.0±1.1 | 0.32 | 0.25 | 39 | 11.4±1.3 | 0.03 | 0.02 | 13 | 11.1±1.3 | 0.71 | 0.73 |
|  | Above median | 4 | 10.3±0.8 |  |  | 40 | 10.8±0.9 |  |  | 22 | 10.9±0.9 |  |  |
| **Abdominal circumference** | TWA |  |  |  |  |  |  |  |  |  |  |  |  |
|  | Below median | 10 | 33.8±1.5 | 0.14 | 0.18 | 39 | 34.1±2.1 | 0.15 | 0.04 | 14 | 33.4±2.2 | 0.93 | 0.59 |
|  | Above median | 4 | 32.1±1.9 |  |  | 40 | 33.4±2.1 |  |  | 21 | 33.4±1.3 |  |  |
|  | P50 |  |  |  |  |  |  |  |  |  |  |  |  |
|  | Below median | 7 | 33.7±1.7 | 0.46 | 0.21 | 36 | 34.1±2.1 | 0.22 | 0.06 | 12 | 33.2±2.5 | 0.68 | 0.33 |
|  | Above median | 7 | 33.0±1.8 |  |  | 43 | 33.5±2.1 |  |  | 23 | 33.5±1.2 |  |  |
|  | P75 |  |  |  |  |  |  |  |  |  |  |  |  |
|  | Below median | 10 | 33.8±1.5 | 0.14 | 0.18 | 39 | 34.1±2.1 | 0.26 | 0.02 | 13 | 33.3±2.4 | 0.82 | 0.30 |
|  | Above median | 4 | 32.1±1.9 |  |  | 40 | 33.5±2.1 |  |  | 22 | 33.4±1.3 |  |  |

^#^ Adjusting for family income, gestational age, parity, parental age at childbirth, parental BMI before pregnancy, and maternal passive smoking during pregnancy.
